# i4mC-EL: Identifying DNA N4-Methylcytosine Sites in the Mouse Genome Using Ensemble Learning

**DOI:** 10.1155/2021/5515342

**Published:** 2021-05-29

**Authors:** Yanjuan Li, Zhengnan Zhao, Zhixia Teng

**Affiliations:** College of Information and Computer Engineering, Northeast Forestry University, Harbin 150040, China

## Abstract

As one of important epigenetic modifications, DNA N4-methylcytosine (4mC) plays a crucial role in controlling gene replication, expression, cell cycle, DNA replication, and differentiation. The accurate identification of 4mC sites is necessary to understand biological functions. In the paper, we use ensemble learning to develop a model named i4mC-EL to identify 4mC sites in the mouse genome. Firstly, a multifeature encoding scheme consisting of Kmer and EIIP was adopted to describe the DNA sequences. Secondly, on the basis of the multifeature encoding scheme, we developed a stacked ensemble model, in which four machine learning algorithms, namely, BayesNet, NaiveBayes, LibSVM, and Voted Perceptron, were utilized to implement an ensemble of base classifiers that produce intermediate results as input of the metaclassifier, Logistic. The experimental results on the independent test dataset demonstrate that the overall rate of predictive accurate of i4mC-EL is 82.19%, which is better than the existing methods. The user-friendly website implementing i4mC-EL can be accessed freely at the following.

## 1. Introduction

As a chemical modification occurring on DNA sequences, DNA methylation can change genetic properties under the condition that the order of DNA sequences remains unchanged. DNA methylation has many manifestations, such as 5-methylcytosine (5mC for short), N6-methyladenine (6 mA for short), and N4-methylcytosine (4mC for short) [1]. Among them, the 5mCs are widely present in prokaryotes and eukaryotes and are of great significance for controlling gene differentiation and gene expression, maintaining chromosome stability and cell structure [2, 3]. They also can cause some diseases such as cancer [4–6]. The 6mAs are also widely distributed in prokaryotes and eukaryotes, which play a crucial role in replication, expression, and transcription of gene [7]. The 4mCs which were found in 1983 mainly exist in prokaryotes, and they can control DNA replication, gene expression, and cell cycle [8]. However, compared with 5mCs and 6mAs, the current research on 4mCs is still insufficient. To make up for this defect and further understand 4mCs' biological properties and functions, the first thing we need to do is to identify 4mCs from various DNA sequences, which is still a hot research topic so far.

In order to identify 4mCs, many biology-based approaches have been explored. Single molecule real-time sequencing technology (SMRT for short) [9, 10] detects optical signals of bases matching the template at the single-molecule level to identify 4mCs. 4mC-Tet-assisted-bisulfite-sequencing technology (4mC-Tet for short) [11] identifies 4mCs by using bisulfite to convert unmethylated cytosine in the DNA sequences into uracil while to keep methylated cytosine unchanged. However, this kind of technologies is time-consuming and resource-intensive. Moreover, the explosive growth of DNA sequences also makes it more difficult to achieve whole-genome sequencing through these technologies. Therefore, using machine learning (ML for short) to identify 4mCs shows more advantages. Up to now, there are many models using machine learning to identify 4mCs. iDNA4mC [12], the earliest model for 4mC identification, is primarily used to identify 4mCs from the genomes of six species, A.thaliana, C.elegans, D.melanogaster, E.coli, G.pickeringii, and G.subterraneus, and its positive data containing 4mCs were obtained from a reliable database called MethSMRT [13]. Soon afterwards, several other models, 4mcPred [14], 4mcPred-SVM [15], 4mcPred-IFL [16], and Meta-4mcPred [17], were proposed successively, which used the same dataset as iDNA4mC [12] for 4mCs identification of the genomes of these six species. i4mC-Rose [18] is the first and the only model for 4mCs identification in the genome of Rosaceae, and it derived positive dataset from the MDR [19] and the other reliable database for storing 4mC data. For the mouse genome we wanted to study, there have been currently two models, 4mCpred-EL [20] and i4mC-Mouse [21]. Among them, their samples containing 4mCs were also obtained from the MethSMRT database. In addition, 4mCpred-EL selected 4 ML algorithms and 7 feature encoding schemes to generate 28 sets of results as the final coding. Subsequently, 4mCpred-EL trained 4 submodels through the final coding and these 4 ML algorithms and then combined the 4 submodels into the final model by majority voting. i4mC-Mouse trained 6 submodels using 6 feature encoding schemes and random forest (RF for short) algorithm, and then the 6 submodels were combined into the final model by weighted voting. Compared with 4mCpred-EL, i4mC-mouse has better performance according to the indicators, ACC and MCC. Although exciting results have been achieved in 4mCpred-EL and i4mC-Mouse, the performance is able to be further increased. In this paper, to further improve the prediction capability, we propose a new mouse's 4mCs predictor, i4mC-EL.

## 2. Materials and Methods

### 2.1. Framework of i4mC-EL

**Figure 1:**
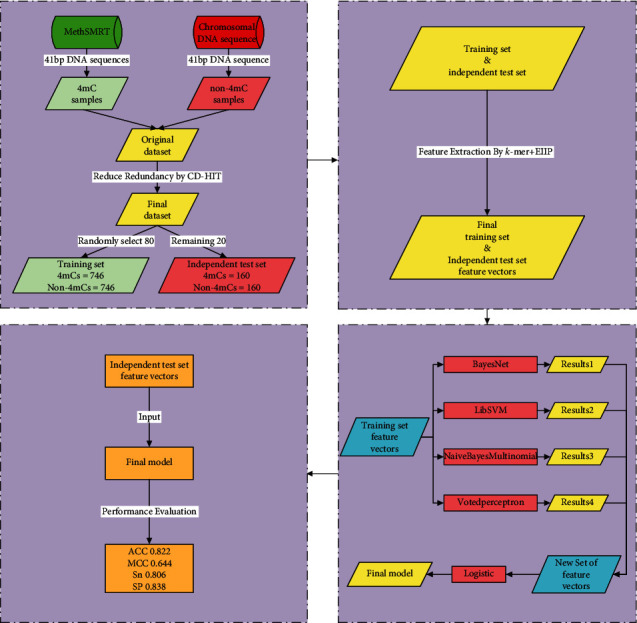
The framework of i4mC-EL.

In the present study, a novel model named i4mC-EL is proposed to indentify mouse's 4mCs, and we can see the framework of it in Figure 1. First, using two different feature encoding schemes, Kmer and EIIP, each DNA sequence was encoded into a 1364-dimensional vector and a 41-dimensional vector, respectively. Next, the 1364-dimensional vector and the 41-dimensional vector of each DNA sequence were combined to form a 1405-dimensional multifeature vector. Finally, a two-stage stacked ensemble learning classifier with these multifeature vectors as input was constructed. The ensemble classifier used BayesNet, NavieBayes Multinomial, LibSVM, and Voted Perceptron as base classifiers and used Logistic as metaclassifier. i4mC-EL's datasets and feature encoding schemes and classifiers will be described detailly below.

### 2.2. Dataset

This paper adopted the benchmark dataset constructed by Hasan'steam [21]. In this dataset, the positive samples containing mouse's 4mCs were obtained from the MethSMRT [17] database, and the negative samples were taken from chromosome DNA sequences. They were all fragments of DNA sequences consisting of 41 nucleotides with a “C” in the middle. Only the sequences whose modQV value greater than or equal to 20 were considered to obtain the high-quality dataset. To prevent the predictor from overfitting, the threshold of CD-HIT [22] was set to 70% to remove redundant sequences [23]. The dataset contained 1,812 DNA sequences, 906 of which were 4mCs and 906 were non-4mCs. About 80% of the dataset was randomly selected as the training dataset, and the remaining about 20% was used as the independent test dataset. The training dataset (train-1492) consisted of 746 4mCs and 746 non-4mCs. And the independent test dataset (test-320) included 160 4mCs and 160 non-4mCs.

### 2.3. Feature Encoding

Transforming DNA sequences into vectors that can make a distinction between 4mCs and non-4mCs availably is the first step to build an ensemble learning-based predictor to identify 4mCs [24–29]. Here, a multifeature encoding scheme composed of Kmer [30–33] and EIIP [34] was used to encode DNA sequences. Kmer represented the DNA sequences as the occurrence frequencies of *k* adjacent nucleotides. EIIP encoded each nucleotide in DNA sequences with its corresponding electron-ion energy. In the experiment of Section 3, we will find that this multifeature is able to encode DNA sequences availably. The following parts are detailed descriptions of Kmer and EIIP.

#### 2.3.1. Kmer

This encoding scheme refers to the frequency of *k*-nucleotides composed of *k* continuous nucleotides in each sequence. For sequence *D* = *d*_1_*d*_2_*d*_3_ ⋯ *d*_*L*−2_*d*_*L*−1_*d*_*L*_, each element of each feature vector is calculated by Equation (1):

(1) $$\begin{array}{c} f\left(X\right)=\frac{F\left(X\right)}{L-k+1}, \end{array}$$

where *X* is one of the *k*-nucleotide, *F*(*X*)and *f*(*X*) are the count and frequency of *X* in *D*, respectively, and *L* is *D*'s length. After Kmer, sequences are transformed into 4^*k*^-dimensional vectors. For example, when the *k*-mer parameter *k* = 2, the value of *AA* in the 16-dimensional (4^2^) feature vector of sequence *D*_1_ = AAACTAGTC is 0.25.

In the present study, we choose the values of the parameter *k* to be 1, 2, 3, 4, and 5, generating 1364-dimensional (4^1^ + 4^2^ + 4^3^ + 4^4^ + 4^5^) feature vectors.

#### 2.3.2. EIIP

EIIP is the short name of electron-ion interaction pseudopotential. The encoding scheme based on EIIP was proposed by Nair and Sreenadhan in 2006. Through it, each nucleotide in each sequence is replaced by its corresponding electron-ion interaction pseud potential value (Table 1). For example, the result of sequence *D*_2_ = AACTG after EIIP encoding is (0.1260, 0.1260, 0.1340, 0.1335, 0.0806). In the present study, each sequence is transformed into a 41-dimensional feature vector.

### 2.4. Classifier

As an open data mining platform, Weka has assembled a large number of machine learning algorithms that can undertake data mining tasks. In the present paper, the classifiers we used were all implemented by Weka, such as BayesNet, NaiveBayes, SGD, SimpleLogistic, SMO, IBk, JRip, J48, and ensemble learning. Finally, we chose the ensemble learning, and the results of related experiment will be presented in section 3.

According to different combination strategies, bagging, boosting, and stacking are the three main types of ensemble learning. Ensemble learning is widely used in bioinformatics because it can improve the prediction performance of classifiers, such as protein-protein interaction [35], disease prediction [36], type III secreted effectors prediction [37], and protein subcellular location prediction [38]. In detail, we used two-stage stacked ensemble learning.

In the two-stage stacked ensemble learning, the base classifiers used in this paper include BayesNet [39], Voted Perceptron [40], Naive Bayes Multinomial [41], and LibSVM [42], and the metaclassifier was Logistic. At the first stage of the ensemble learning classifier, based on the multifeature vectors proposed in this paper, four base classifiers are, respectively, trained to relabel the training dataset and the independent test dataset. At the second stage, the outputs of base classifiers are utilized as input for the metaclassifier.

**Figure 2:**
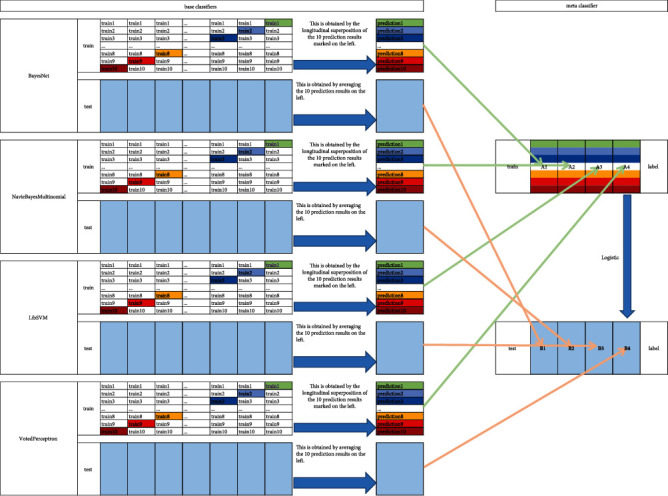
Working diagram of ensemble learning.

Figure 2 gives the detailed process of model generation and result output, the steps are as follows.

**Step 1 .**

Partition dataset. Divide the training dataset into ten parts and mark them as train 1, train 2,…, train 10. The independent test dataset remains unchanged.

**Step 2 .**

Train base classifiers. In the present paper, we chose BayesNet, Voted Perceptron, Naive Bayes Multinomial, and LibSVM as base classifiers. For one base classifier such as BayesNet, 10-fold crossvalidation is performed. In detail, train 1, train 2,…, train 10 are used as validation dataset in turn, the other nine parts are used as the training dataset, and prediction is made on the independent test dataset. This would get 10 predictions from the training dataset together with another 10 predictions on the independent test dataset. Combine the 10 predictions on the training dataset vertically to get A1 and take the average of the 10 predictions on the independent test dataset to get B1. Similarly, we could get A2, B2 from NavieBayes Multinomial, A3, B3 from LibSVM, and A4, B4 from Voted Perceptron.

**Step 3 .**

Train metaclassifiers. Use the predictive values of the 4 base classifiers on the training dataset, A1, A2, A3, and A4, as 4 features to train the logistic classifier.

**Step 4 .**

Predict new data. Use the trained model to make predictions on the 4 features, B1, B2, B3, and B4, constructed from the predicted values of the independent test dataset of the 4 base classifiers, and then the final prediction results are obtained.

### 2.5. Performance Evaluation

For the sake of validating the quality of our classification predictor, we used four indicators widely adopted in the field of bioinformatics for evaluation [43–53]. These indicators can be calculated using the formulas below:

(2) $$\begin{array}{c} \text{ACC}=\frac{\text{TN}+\text{TP}}{\text{TN}+\text{FN}+\text{FP}+\text{TP}},\\ \text{MCC}=\frac{\text{TN}\times \text{TP}-\text{FN}\times \text{FP}}{\sqrt{\left(\text{TN}+\text{FN}\right)\times \left(\text{FN}+\text{TP}\right)\times \left(\text{TP}+\text{FP}\right)\times \left(\text{FP}+\text{TN}\right)}},\\ \text{Sn}=\frac{\text{TP}}{\text{FN}+\text{TP}},\\ \text{Sp}=\frac{\text{TN}}{\text{FP}+\text{TN}}, \end{array}$$

where TP indicates the number of the sequences that they are actually 4mCs, and that they are identified as 4mCs by the model, FP indicates the number of the sequences that they are actually non-4mCs but that they are identified as 4mCs by the model, TN indicates the number of the sequences that they are actually non-4mCs, and that they are identified as non-4mCs by the model, FN indicates the number of the sequences that they are actually 4mCs but that they are identified as non-4mCs by the model. The Sn refers to the prediction accuracy of 4mCs. The Sp refers to the prediction accuracy of non-4mCs. ACC refers to the prediction accuracy of both 4mCs and non-4mCs. MCC represents the reliability of the prediction results. The higher the values of the above four indicators have, the more superior the capability of the predictor would be.

## 3. Results and Discussion

### 3.1. Crossvalidation Results of TRAIN-1492

To find the features that can adequately represent the structure and function of the DNA sequences, we attempted to contrast numerous feature encoding schemes. And to achieve the optimal accuracy, we also tried to train the model using several different classification algorithms. The results of relevant comparative experiments are as below.

#### 3.1.1. Feature Encoding Comparison on Crossvalidation

AS shown in section of “feature encoding,” we encode the DNA sequences with a multifeature, which combines *k*-mer and EIIP feature encoding method. To verify the validity of the proposed multifeature, we compare the proposed multifeature with BPF, DPE, RFHC, RevKmer, and PseKNC feature encoding schemes and their combinations using ensemble learning classification. Among them, BPF and DPE are encoding schemes based on nucleotide positions, in which BPF takes mononucleotides as its encoding targets, while DPE takes dinucleotides as its encoding targets. RFHC is an encoding scheme based on the physicochemical properties of nucleotides. RevKmer is a variant of Kmer that considers not only the current *k*-nucleotides themselves, but also their reverse complementary nucleotides. PseKNC is a method to integrate continuous local and global *k*-tuple nucleotide information into the feature vectors of DNA sequences.

Table 2 displays experimental results, in which “our method” denotes the multifeature mentioned in the section “feature encoding.” As shown in Table 2, from the perspective of ACC and MCC, the index values of our method are higher than those of all other feature encoding schemes, which indicates that our method has a better overall performance. From the perspective of Sp, the index value of our method is still the highest, which indicates that it is more dominant to identify non-4mC from negative samples. These conclusions demonstrate that our method has good validity.

**Figure 3:**
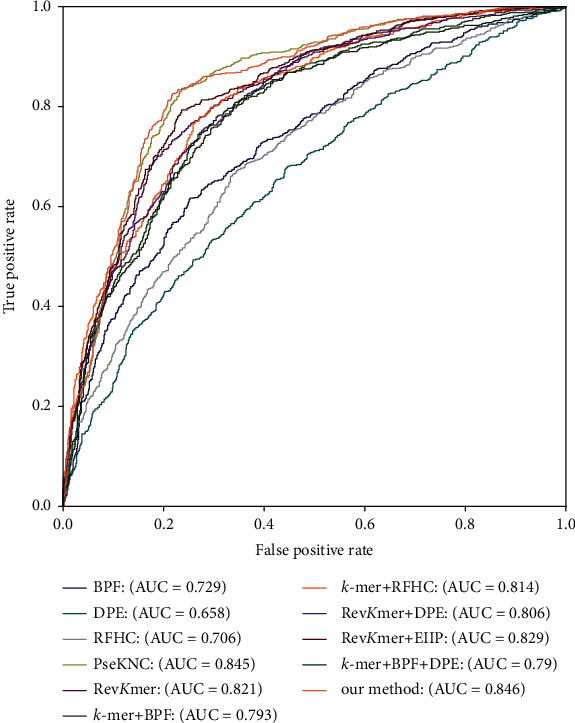
ROC curves for dissimilar feature encoding schemes under 10-fold crossvalidation.

To further illustrate the prediction capability of our selected multifeature encoding scheme, the ROC curves for dissimilar feature encoding schemes under 10-fold crossvalidation are displayed in Figure 3. From Figure 3, we can see that our method has the largest area under ROC curve (AUC), which demonstrates that our method can represent mouse's DNA sequences better than others.

#### 3.1.2. Classifier Comparison on Crossvalidation

As shown in the section “classifier,” we inputted the multifeature composed of *k*-mer and EIIP into an ensemble learning classifier called stacking, then obtained a predictor which is used for identifying mouse's 4mCs. To verify the validity of stacking used in this paper, on the basis of the multifeature used in this paper, we compared stacking with eleven commonly used classifiers, BayesNet, Naive Bayes, SGD, Simple Logistic, SMO, IBK, JRip, J48, Random Forest, AdaBoostM1, and Bagging. Among them, BayesNet characterizes the dependencies among attributes with the aid of directed acyclic graphs and uses conditional probability tables to describe the joint probability distribution of attributes. NaiveBayes is a simple probabilistic classifier based on Bayes' theorem under the assumption that each attribute is independent of each other. SGD implements a regularized linear support vector machine classifier with stochastic gradient descent learning. Simple Logistic is a linear logistic regression classifier with only one independent variable. SMO is a support vector machine classifier using a continuous minimum optimization algorithm. IBk classifies the data point by determining the category of *k* data points closest to it. JRip is a classifier based on rule induction. J48 is a decision tree classifier that uses information gain rate to select attributes for partitioning. Random Forest refers to a classifier that utilizes multiple trees to train and predict a sample. AdaBoostM1 is a classifier that enables the previously incorrectly predicted training samples to receive more attention at follow-up by adjusting their distribution. Bagging uses bootstrap sampling to obtain m (m is the predetermined number of base classifiers) sample datasets from the original dataset, which are used to train *m* base classifiers that are then integrated by voting.

The results of these comparative experiments are displayed in Table 3, where “our method” refers to the stacking classifier. From Table 3, we can see that our method outperforms the other classifiers in all indicators.

**Figure 4:**
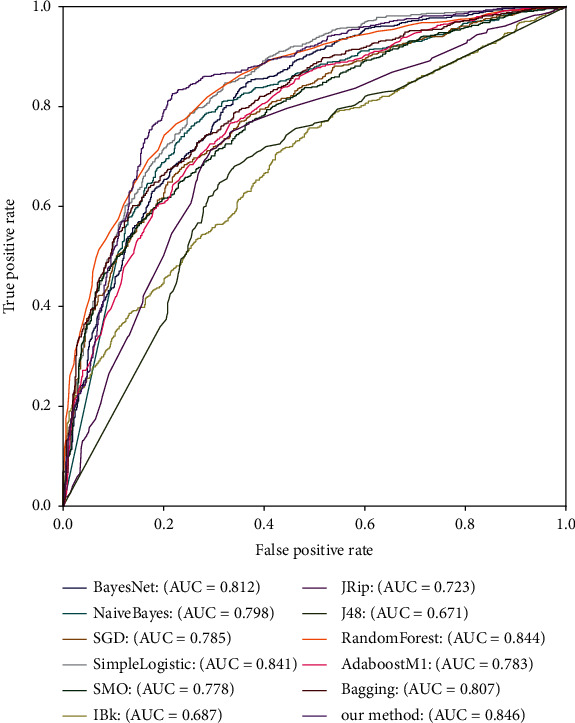
ROC curves for dissimilar classifiers under 10-fold crossvalidation.

To further illustrate the classification capability of our selected stacking classifier, the ROC curves for dissimilar classifiers under 10-fold crossvalidation are displayed in Figure 4. From Figure 4, we can see that the area under ROC curve (AUC) of our method is the largest, which proves that our proposed method has better prediction performance for identifying 4mCs in the mouse genome than other methods.

### 3.2. Independent Validation Results of TEST-320

In this section, a comparative experiment on the independent test dataset (TEST-320) will be conducted to show the generalization capability of our selected multifeature and stacking classifier. The rationale for this is that this model is trained and tested on two different datasets, which is the equivalent of performing a real prediction task with the generated model.

#### 3.2.1. Feature Encoding Comparison on Independent Validation

Using the stacking classifier, we, respectively, evaluate the generalization capability of various feature encoding schemes described in Section 3.1.1 on TEST-320.  Table 4 displays these comparison experimental results. From Table 4, among the compared feature encoding schemes, our method performed best in ACC, Sn, and MCC, which were 82.19%, 0.806, and 0.644, respectively. Although the Sp of our method is lower than that of BPF, *k*-mer + BPF, *k*-mer + RFHC, *k*-mer + BPF + DPE, and PseKNC+EIIP+RFHC, the other three indicators of our method are higher than theirs.

**Figure 5:**
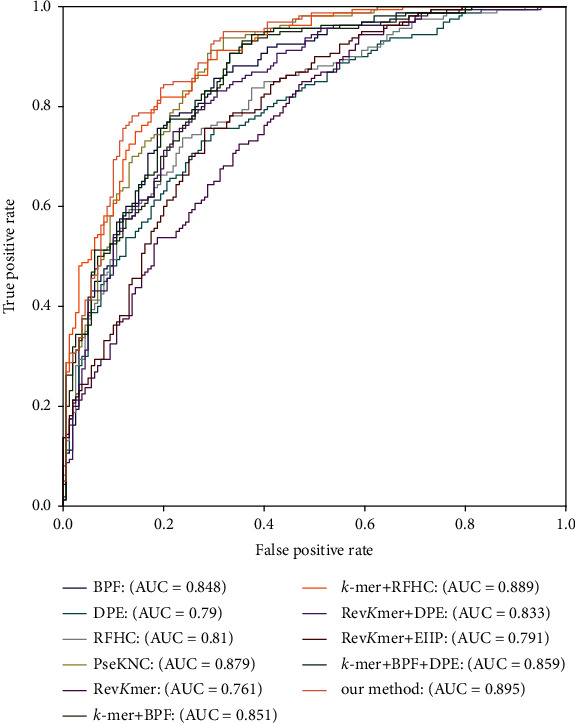
ROC curves for dissimilar feature encoding schemes on TEST-320.

For the sake of further describing the generalization capability of our selected multifeature encoding scheme, Figure 5 displays the ROC curves for dissimilar feature encoding schemes on TEST-320. From Figure 5, we can see that the AUC of our method is the largest, and the ROC curve of our method is closer to the upper left, which demonstrates that our selected multifeature is more suitable than other schemes to encode the DNA sequences used to recognize mouse's 4mC.

#### 3.2.2. Classifier Comparison on Independent Validation

We compared stacking classifier used in this paper with other eleven classifiers on TEST-320 under the condition of using the multifeature combing *k*-mer and EIIP as the input of the stacking. The results of these comparative experiments are displayed in Table 5, from which we can see that although the Sp of BayesNet is a little higher than that of our method, our method outperforms other classifiers in ACC, Sn, and MCC. Overall, our selected stacking classifier performs better than the others, indicating that it is effective for identifying mouse's 4mC.

**Figure 6:**
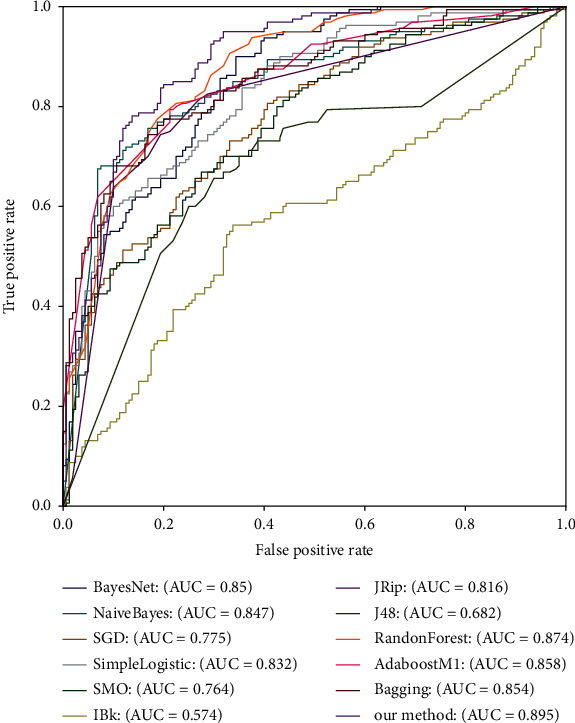
ROC curves for dissimilar classifiers on TEST-320.

For the sake of further describing the generalization capability of our selected stacking classifier, the ROC curves for dissimilar classifiers on TEST-320 are displayed in Figure 6, where we can get the conclusion that the AUC of our method is the largest too, which proves that our proposed stacking-based ensemble classifier method is more suitable for the identification of mouse's 4mCs than other classifiers.

### 3.3. Contrast with Extant Models on TEST-320

Here, we contrasted i4mC-EL with 4mCpred-EL and i4mC-Mouse on TEST-320 for the sake of further evaluating its performance.  Table 6 displays these contrast experimental results, in which the data of 4mCpred-EL and i4mC-Mouse are from reference. From Table 6, we can see that i4mC-EL is superior to 4mcPred-EL and i4mC-Mouse in three indexes which are ACC, Sp, and MCC. Although the Sn of i4mC-Mouse is a little higher than that of our method, our method outperforms i4mC-Mouse in the other three indexes. All in all, i4mC-EL performs better than extant methods.

## 4. Conclusions

In the present paper, an ensemble learning model called i4mC-EL which was able to identify mouse's 4mC sites was designed. In the process of constructing i4mC-EL, to determine the optimal combination of feature encoding schemes and classifiers, we conducted abundant comparative experiments on dissimilar features and classifiers. Finally, we encoded DNA sequences with multifeatures combing *k*-mer and EIIP, then used two-stage stacked ensemble learning as classifier. We used BayesNet, NavieBayes Multinomial, LibSVM, and VotedPerceptron as base classifiers and Logistic as metaclassifier.

In addition, we contrasted i4mC-EL with existing models for the sake of proving its effectiveness. The results show that i4mC-EL is better than the existing models and has better generalization capability. In summary, i4mC-EL is effective in predicting the 4mC sites in the mouse genome, which helps us to understand the biochemical properties of 4mC.

We will use adaptive feature vectors to donate DNA sequences to optimize the feature encoding scheme [54, 55] in the future work. Furthermore, other improvements, encoding schemes, classifier algorithms, and intelligent computing models to identify 4mC sites will also be considered.

## Figures and Tables

**Table 1:** The electron-ion interaction pseudopotential values for DNA nucleotides.

NT	A	C	G	T
EIIP	0.1260	0.1340	0.0806	0.1335

**Table 2:** The contrast of performance for dissimilar feature encoding schemes under 10-fold crossvalidation.

Schemes	ACC	MCC	Sn	Sp
BPF	0.668	0.335	0.665	0.670
DPE	0.614	0.228	0.619	0.609
RFHC	0.658	0.316	0.669	0.647
RevKmer	0.755	0.511	0.745	0.765
PseKNC	0.794	0.589	0.786	0.803
*k*-mer + BPF	0.724	0.448	0.729	0.718
*k*-mer + RFHC	0.747	0.493	0.744	0.749
RevKmer+DBE	0.738	0.476	0.723	0.753
RevKmer+EIIP	0.779	0.558	0.764	0.794
*k*-mer + BPF + DPE	0.732	0.464	0.741	0.723
Our method	0.803	0.606	0.784	0.822

**Table 3:** The contrast of performance for dissimilar classifiers under 10-fold crossvalidation.

Classifiers	ACC	MCC	Sn	Sp
BayesNet	0.727	0.453	0.739	0.714
NaiveBayes	0.752	0.504	0.751	0.753
SGD	0.712	0.424	0.710	0.713
SimpleLogistic	0.761	0.522	0.753	0.768
SMO	0.702	0.405	0.706	0.698
IBk	0.637	0.276	0.584	0.690
JRip	0.707	0.414	0.692	0.723
J48	0.665	0.330	0.674	0.655
RandomForest	0.770	0.541	0.753	0.787
AdaBoostM1	0.713	0.427	0.739	0.688
Bagging	0.729	0.459	0.744	0.714
Our method	0.803	0.606	0.784	0.822

**Table 4:** The contrast of performance for dissimilar feature encoding schemes on TEST-320.

Schemes	ACC	MCC	Sn	Sp
BPF	0.753	0.530	0.606	0.900
DPE	0.697	0.401	0.600	0.794
RFHC	0.716	0.438	0.631	0.800
RevKmer	0.666	0.335	0.744	0.588
PseKNC	0.781	0.563	0.788	0.775
*k*-mer + BPF	0.772	0.553	0.681	0.863
*k*-mer + RFHC	0.800	0.614	0.694	0.906
RevKmer+DBE	0.756	0.516	0.700	0.813
RevKmer+EIIP	0.713	0.427	0.763	0.663
*k*-mer + BPF + DPE	0.772	0.553	0.681	0.863
Ourmethod	0.822	0.644	0.806	0.838

**Table 5:** The contrast of performance for dissimilar classifiers on TEST-320.

Classifiers	ACC	MCC	Sn	Sp
BayesNet	0.769	0.547	0.675	0.863
NaiveBayes	0.788	0.577	0.744	0.831
SGD	0.688	0.379	0.756	0.619
Simple Logistic	0.728	0.456	0.738	0.719
SMO	0.675	0.353	0.744	0.606
IBk	0.600	0.201	0.563	0.638
JRip	0.769	0.541	0.713	0.825
J48	0.663	0.325	0.656	0.669
Random Forest	0.778	0.558	0.738	0.819
AdaBoostM1	0.791	0.581	0.794	0.788
Bagging	0.781	0.564	0.744	0.819
Our method	0.822	0.644	0.806	0.838

**Table 6:** The contrast of performance for dissimilar models on TEST-320.

Models	ACC	MCC	Sn	Sp
4mcPred-EL	0.791	0.584	0.757	0.825
i4mC-Mouse	0.816	0.633	0.807	0.825
i4mC-EL	0.822	0.644	0.806	0.838

## Data Availability

The datasets used during the present study are available from the corresponding author upon reasonable request, or can be downloaded from http://106.12.83.135:8080/i4mC-EL/.
